# Active surveillance of highly suspicious thyroid nodules in the isthmus compared with non-isthmus locations: a multicenter retrospective study

**DOI:** 10.3389/fendo.2026.1830199

**Published:** 2026-05-07

**Authors:** Yan Hu, Wei Zhou, Cheng Li, Lu Zhang, Weiwei Zhan

**Affiliations:** 1 Department of Ultrasound, Ruijin Hospital, Shanghai Jiaotong University School of Medicine, Shanghai, China; 2 Faculty of Medical Imaging Technology, College of Health Science and Technology, Shanghai Jiao Tong University School of Medicine, Shanghai, China; 3 Department of Ultrasound, Ruijin Hospital Lu Wan Branch, Shanghai Jiao Tong University School of Medicine, Shanghai, China; 4 Department of Ultrasound, Ruijin Hospital Jiading Branch, Shanghai Jiao Tong University School of Medicine, Shanghai, China

**Keywords:** active surveillance (AS), isthmus, papillary thyroid carcinoma, suspicious thyroid nodules, ultrasound

## Abstract

**Objectives:**

To compare progression outcomes during active surveillance between highly suspicious thyroid nodules located in the isthmus and those in non-isthmic regions, and to explore the feasibility of active surveillance for selected isthmic lesions.

**Methods:**

This multicenter retrospective study included 449 solitary thyroid nodules that were re-evaluated by blinded image review and classified as American College of Radiology Thyroid Imaging Reporting and Data System (ACR TI-RADS) category 5. Nodules were anatomically categorized as isthmic (n = 33) or non-isthmic (n = 416). Cytological results were available for 144 of 449 nodules (32.1%). Progression was evaluated according to size-based criteria (≥3 mm increase in maximum diameter, ≥3 mm increase in one, two, or all three orthogonal diameters, and ≥50% increase in volume), the occurrence of new suspicious lesions, lymph node metastasis, and distant metastasis, as well as a composite endpoint encompassing the latter three events.

**Results:**

The median follow-up duration was 47 months, with no significant difference between the two groups. Non-isthmic nodules had significantly larger volumes at baseline and at final follow-up, whereas maximum diameter did not differ significantly between groups (P>0.05). A ≥50% increase in volume occurred in 30.30% of the isthmus group and 24.52% of the non-isthmus group (P = 0.53). A composite endpoint occurred in 3.03% of the isthmus group and 4.09% of the non-isthmus group (P = 1.00). Observed differences in progression indicators were not statistically significant (P>0.05). No distant metastases or thyroid cancer-related deaths occurred during follow-up.

**Conclusion:**

In this multicenter retrospective cohort, ACR TI-RADS 5 thyroid nodules located in the isthmus were not observed to have higher progression rates than non-isthmic nodules during AS. These findings support the potential feasibility of active surveillance for selected isthmic nodules under careful monitoring. However, the small isthmus sample size and low number of progression events preclude definitive conclusions regarding equivalence between the two groups.

## Introduction

The prevalence of thyroid nodules is high, with papillary thyroid carcinoma (PTC) accounting for approximately 80% of malignant thyroid tumors (1, 2). Surgical resection remains the standard treatment for PTC (3). However, for selected low-risk cases without lymph node metastasis (LNM), both thermal ablation and active surveillance (AS) have demonstrated comparable safety and efficacy (4–7). Fine-needle aspiration (FNA) is commonly required to confirm malignancy prior to surgery or ablation. Yet, the necessity of FNA prior to initiating AS remains debated, particularly in patients who prefer a non-invasive management strategy. For these patients, the willingness to accept potential malignancy in favor of long-term monitoring often supersedes the diagnostic information provided by FNA, which rarely alters the decision to delay intervention. Recent expert consensus has acknowledged this evolving paradigm, suggesting that AS without prior FNA may be appropriate for highly suspicious nodules if patients are adequately informed and compliant with close follow-up (8, 9).

With the increasing global adoption of AS, clinical management of PTC in China is also evolving. An increasing number of patients with suspicious thyroid nodules are opting for AS to avoid invasive diagnostics and definitive treatments. However, concerns remain regarding the safety of AS in certain anatomical subgroups—particularly in nodules located within the thyroid isthmus.

The isthmus is anatomically narrow, thinly encapsulated, and in close proximity to the trachea and anterior strap muscles, raising theoretical concerns about early capsular invasion and extrathyroidal extension (10–14). Some studies have associated isthmic tumors with higher risks of LNM and aggressive behavior (15), leading to the historical exclusion of isthmic nodules from AS protocols. Nevertheless, several recent studies on thermal ablation and surgical outcomes have shown no significant differences in progression or local recurrence rates between isthmic and non-isthmic nodules (16–20), challenging the assumption that isthmic location is inherently high-risk.

Despite these findings, there is a lack of dedicated research evaluating the long-term outcomes of suspicious thyroid nodules located in the isthmus under AS. As such, the clinical feasibility of AS in this subgroup remains unclear. The American College of Radiology Thyroid Imaging Reporting and Data System (ACR TI-RADS) is a widely adopted ultrasound-based risk stratification system, increasingly utilized across diverse clinical settings (21). Although variations exist among classification systems such as K-TIRADS (22), EU-TIRADS (23), and C-TIRADS (24), they are all fundamentally grounded in the assessment of sonographic features associated with malignancy risk.

Given these considerations, the present study aims to evaluate the safety and feasibility of AS in patients with highly suspicious thyroid nodules (ACR TI-RADS 5) by comparing disease progression between nodules located in the isthmus and those in non-isthmic regions. By addressing this gap, we aim to refine AS indications and inform individualized management strategies for patients with isthmic thyroid nodules.

## Methods

### Patients and ethics

**Figure 1:**
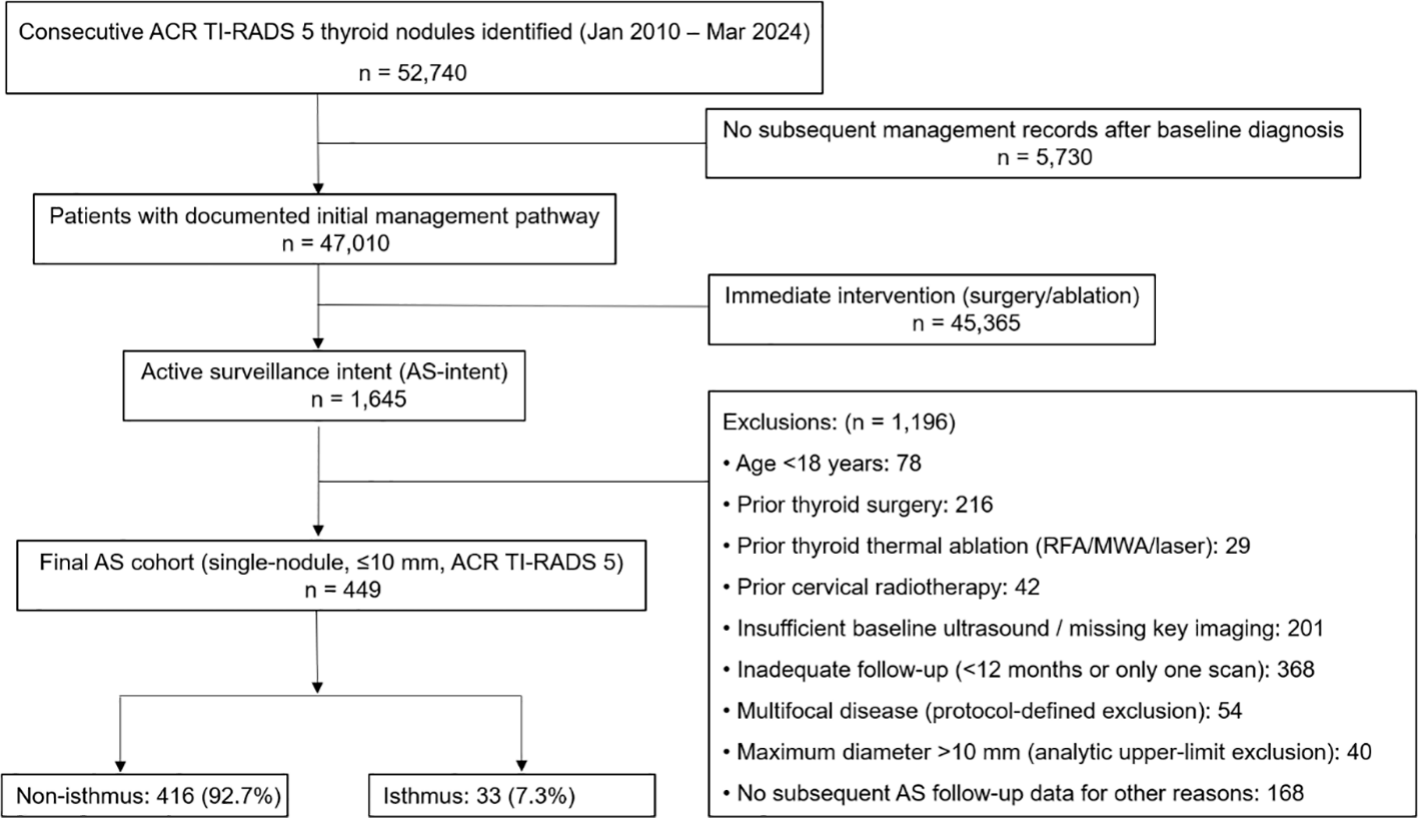
Flowchart of patient selection and grouping. ACR TI-RADS, The American College of Radiology Thyroid Imaging Reporting and Data System.

This retrospective multicenter cohort study was approved by the Ethics Committees of Shanghai Ruijin Hospital (No. 2025122). The requirement for informed consent was waived due to the retrospective design. All methods were carried out in accordance with the Declaration of Helsinki and relevant institutional and national guidelines and regulations. Between January 2010 and March 2024, consecutive patients with ACR TI-RADS 5 thyroid nodules who initially elected AS were screened at the three centers. After applying all inclusion and exclusion criteria (**Figure 1**), 449 patients were included: 359 (79.9%) from Ruijin Hospital, 68 (15.1%) from the Jiading Branch, and 22 (4.9%) from the Luwan Branch. All centers adopted standardized imaging protocols and shared the same electronic medical record and PACS systems to ensure consistency in data acquisition and follow-up evaluation.

### Grouping criteria

**Figure 2:**
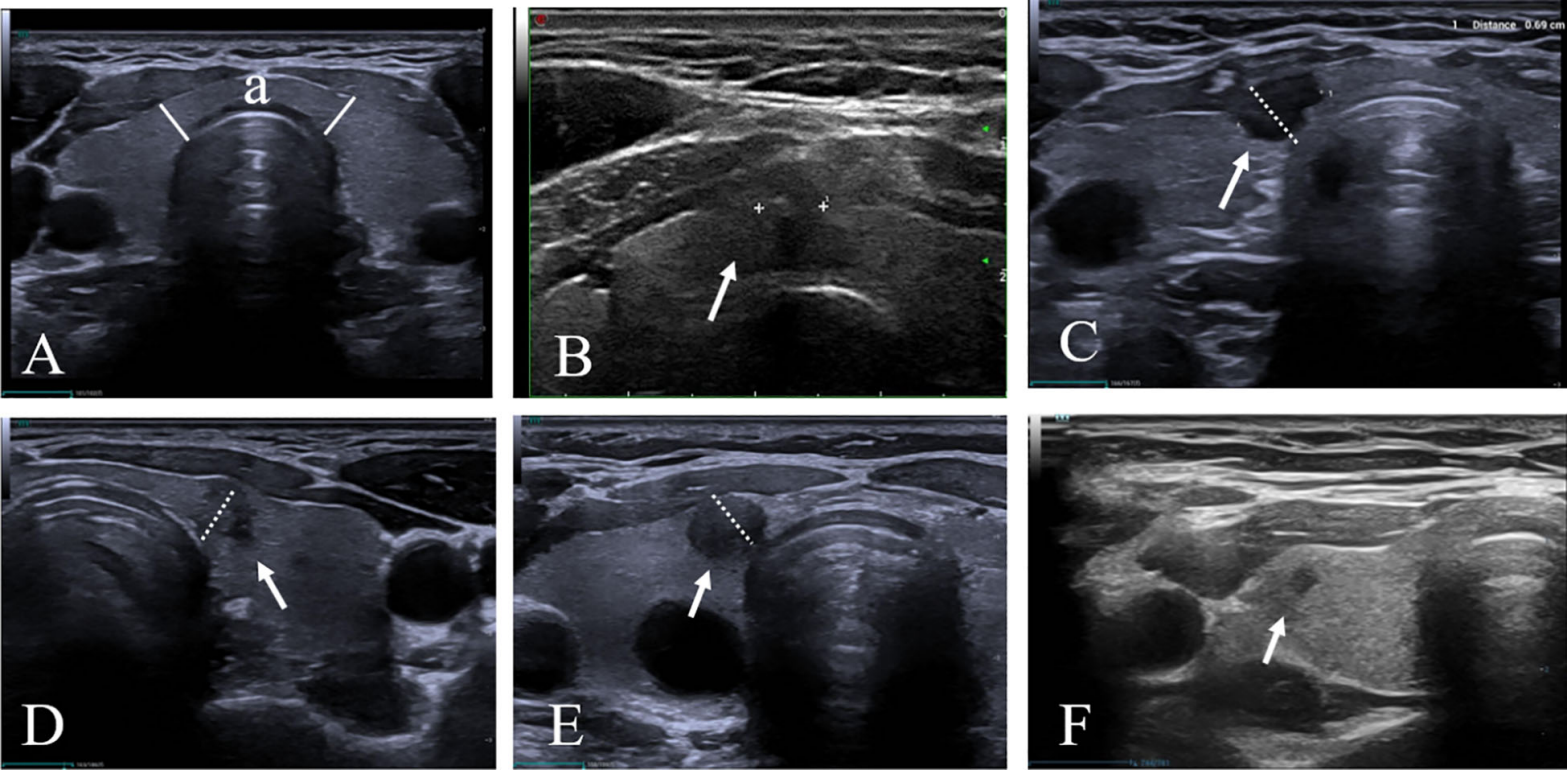
Ultrasound-based definition of isthmic versus non-isthmic thyroid nodules. **(A)** The anterior contour of the trachea was divided into a curved portion and a relatively straight segment using two solid white lines, defining zone a as the isthmic region. **(B, C)** Nodules with more than 50% of their cross-sectional area located within zone a were classified as isthmic. **(D–F)** Nodules located predominantly outside this region were classified as non-isthmic. White arrows indicate the target nodules. White dashed lines represent reference boundaries used to assess whether more than 50% of the nodule lies within zone a.

In this study, the classification of isthmic versus non-isthmic thyroid nodules was primarily based on transverse ultrasound images. Given the lack of a universally accepted sonographic definition of the thyroid isthmus for surveillance-based studies, our operational criteria were developed according to previously published literature and institutional imaging experience in order to provide a pragmatic and reproducible framework for retrospective image review (17, 19, 25, 26).As illustrated in **Figure 2A**, two solid white lines were used to divide the anterior contour of the trachea into a more curved portion and a relatively straight segment. The region between these two lines, referred to as “zone a,” was considered the isthmic zone. Nodules were classified as isthmic if more than 50% of their cross-sectional area was located within this region (**Figures 2B, C**), whereas lesions located predominantly outside zone a were categorized as non-isthmic (**Figures 2D–F**).

### Ultrasound examination and ultrasound image assessment

All thyroid ultrasound examinations were performed using high-resolution linear transducers operating at 7.5–15 MHz. Scanning parameters were optimized based on nodule depth and patient body habitus. All examinations were conducted by board-certified radiologists with at least 3 years of experience in thyroid imaging. According to institutional protocols and international guidelines, patients undergoing AS typically received thyroid ultrasound every 6 months during the first two years and annually thereafter if no progression was observed.

Given the extended diagnostic time span, different ultrasound classification systems were used in routine clinical practice across the study period. Assessments were initially based on the American Thyroid Association (ATA) guidelines, followed by ACR TI-RADS. For consistency in analysis, all nodules were retrospectively reviewed and reclassified according to the ACR TI-RADS. Two board-certified radiologists, each with over 3 years of experience in thyroid imaging, independently performed the retrospective ultrasound assessments. The reviewers were blinded to clinical, cytological, and follow-up data. Only static B-mode images from the initial ultrasound were reviewed using the institutional PACS. Discrepancy was defined as disagreement in the final ACR TI-RADS category, regardless of the specific sonographic features involved. In such cases, a third senior radiologist (>10 years of experience) adjudicated the final classification.

### Ultrasound-based variables and definitions

**Figure 3:**
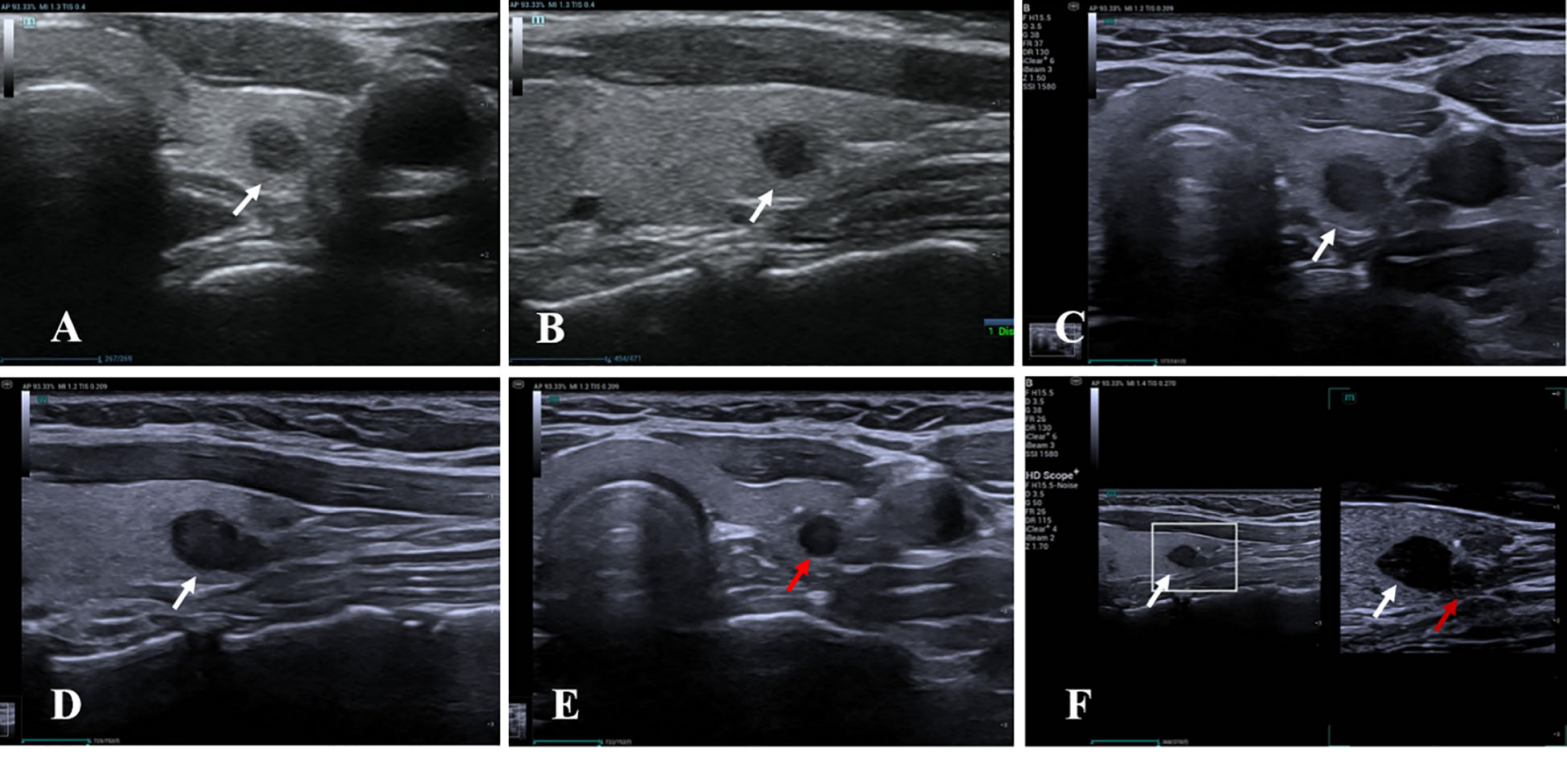
Development of a new suspicious lesion during active surveillance without significant primary nodule growth. A 37-year-old woman was initially diagnosed with a ACR TI-RADS 5 nodule. Baseline transverse **(A)** and longitudinal **(B)** ultrasound images show a suspicious nodule measuring 4.0 × 3.9 × 3.3 mm. At the 18-month follow-up, transverse **(C)** and longitudinal **(D)** images show mild growth of the original nodule to 6.3 × 5.1 × 5.4 mm; however, the increase did not exceed the 3 mm threshold in any single axis. Meanwhile, a new suspicious lesion measuring 2.3 × 3.2 × 3.7 mm appeared inferior to the original nodule. Transverse view **(E)** demonstrates the new lesion, while high-resolution longitudinal imaging **(F)** shows the spatial relationship between the original and new nodules. White arrows indicate the original nodule; red arrows indicate the newly developed lesion.

**Figure 4:**
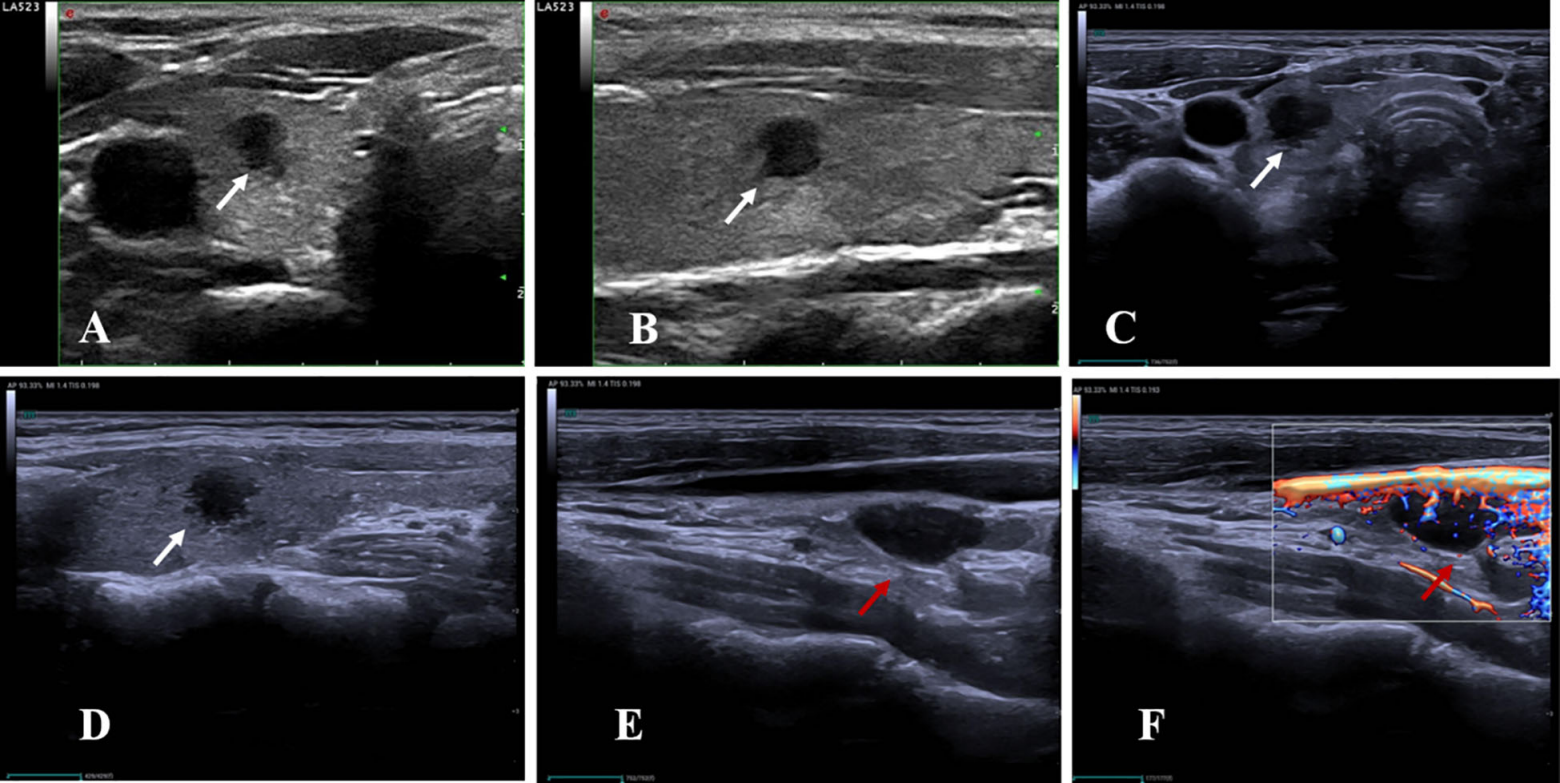
Lymph node metastasis and meeting the ≥3 mm maximum diameter progression criterion during active surveillance of a highly suspicious thyroid nodule. A 35-year-old woman was initially diagnosed with a ACR TI-RADS 5 thyroid nodule. Baseline transverse **(A)** and longitudinal **(B)** ultrasound images show a suspicious nodule measuring 3.9 × 3.9 × 4.3 mm. At the 23-month follow-up, transverse **(C)** and longitudinal **(D)** images demonstrate notable growth of the original nodule to 7.0 × 5.8 × 7.1 mm, meeting the ≥3 mm maximum diameter progression threshold. A suspicious lymph node was simultaneously identified **(E)**, with minimal intranodal vascularity seen on color Doppler imaging **(F)**. Fine-needle aspiration confirmed the diagnosis of lymph node metastasis. White arrows indicate the original thyroid nodule; red arrows indicate the metastatic lymph node.

**Figure 5:**
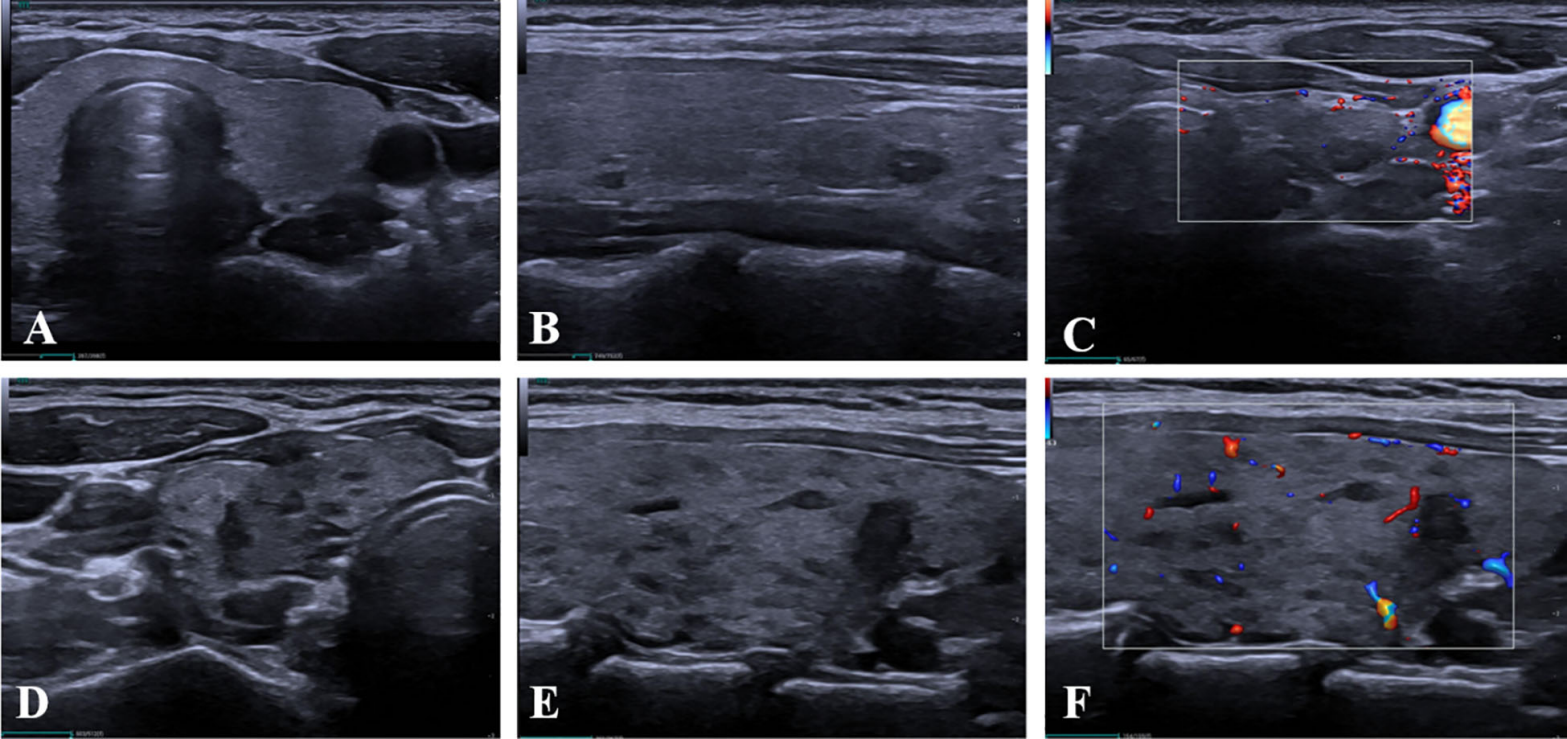
Representative ultrasound criteria for the diagnosis of Hashimoto’s thyroiditis (HT) in this study. **(A–C)** Thyroid nodules without coexisting HT. Gray-scale ultrasound images of the transverse **(A)** and longitudinal **(B)** planes demonstrate a homogeneous parenchymal echotexture. **(C)** Color Doppler imaging reveals minimal intraparenchymal vascularity. **(D–F)** Thyroid nodules with coexisting HT. Gray-scale ultrasound images of the transverse **(D)** and longitudinal **(E)** planes show an inhomogeneous echotexture with diffusely decreased echogenicity and patchy hypoechoic areas. **(F)** Color Doppler imaging demonstrates a moderate degree of intraparenchymal vascular signals.

The following ultrasound variables were assessed: longitudinal location (upper, middle, or lower pole), ACR TI-RADS classification, changes in diameter and volume, follow-up duration, new nodule development (**Figure 3**), LNM (**Figure 4**), and distant metastasis. Hashimoto’s thyroiditis (HT) was included due to its reported association with PTC progression (27, 28). HT was defined sonographically by diffuse parenchymal heterogeneity, with or without glandular enlargement or pseudonodular changes. Representative ultrasound images of nodules with and without HT are shown in **Figure 5**.

### Ultrasound-guided FNA and Bethesda-based management

The decision to perform FNA for highly suspicious thyroid nodules was typically made through shared decision-making between the physician and the patient. Physicians’ recommendations were primarily guided by current clinical guidelines, including those of the ATA and the ACR TI-RADS framework. FNA was performed using a 25-27gauge needle, with at least two passes per thyroid nodule and suspicious lymph node. An additional pass was performed for *BRAF* and *TERT* mutation testing when indicated. Diagnostic results were interpreted according to the Bethesda classification system (29). Patients with Bethesda III or IV nodules were considered for AS if they lacked high-risk clinical or sonographic features. Those with Bethesda V or VI nodules were generally referred for surgery or ablation, unless they opted for AS following thorough counseling.

### Definition of progression events and clinical decision pathways during AS

During active surveillance, progression was evaluated using both size-based indicators and event-based outcomes. Size-based indicators included a ≥3 mm increase in maximum diameter, based on the largest of the three orthogonal diameters measured by ultrasound, a ≥50% increase in volume from baseline to final follow-up, and increases of ≥3 mm in one, two, or all three orthogonal diameters. Nodule volume was calculated using the ellipsoid formula: V = A × B × C × π/6, where A, B, and C represent the three orthogonal diameters. These size-based changes were analyzed separately as individual progression indicators because they may be more susceptible to measurement variability and may not, in isolation, mandate a change in management.

Event-based outcomes included newly developed suspicious lesions, lymph node metastasis, and distant metastasis. Newly developed suspicious lesions were defined as the appearance of new nodules with an ACR TI-RADS classification of 4 or higher, either ipsilateral or contralateral, during follow-up. Lymph node metastasis was defined as nodal metastasis confirmed by FNA or post-surgical pathology. Distant metastasis was defined as disease spread to distant organs, such as the lungs, bones, brain, or liver, confirmed by imaging or pathology. A composite endpoint was defined as the occurrence of any of these three event-based outcomes and was intended to capture progression that was more likely to be clinically actionable during active surveillance.

During follow-up, when clinically actionable progression indicators were identified, particularly newly developed suspicious lesions or suspicious lymph node findings, physicians typically recommended further evaluation or management, such as diagnostic FNA, thermal ablation, or surgery. Size-based changes were interpreted in conjunction with the overall imaging context, serial follow-up findings, and patient preference. In cases where lymph nodes exhibited suspicious features, such as calcification, cystic changes, or echogenicity similar to that of the primary lesion, FNA was generally recommended to evaluate for possible nodal involvement.

### Statistical analysis

All statistical analyses were performed using Python 3.10 (SciPy v1.11) and Pandas libraries. Continuous variables were summarized as mean ± standard deviation (SD). The normality of distribution was assessed using the D’Agostino and Pearson omnibus test. Between-group comparisons of continuous variables were performed using Welch’s t-test in cases of unequal variances (e.g., maximum diameter), or Mann-Whitney U test when normality was violated (e.g., mean volume). Categorical variables were expressed as counts and percentages. Pearson’s chi-square test was used for multi-category comparisons when expected frequencies in each cell were sufficient (e.g., distribution of ACR TI-RADS and Bethesda categories). Fisher’s exact test was applied to analyze 2×2 contingency tables with small sample sizes or rare events (e.g., *BRAF* mutation status). Each progression-related variable (e.g., nodule enlargement, new lesion occurrence, and lymph node metastasis) was considered an independent binary outcome and compared separately using Fisher’s exact test. Because multiple individual progression indicators were evaluated in addition to the composite endpoint, p-values for individual indicators were considered exploratory and were interpreted descriptively to characterize progression patterns rather than to establish definitive statistical significance. For the principal binary progression outcomes, 95% confidence intervals were calculated for group-specific proportions using the Clopper-Pearson exact method, and 95% confidence intervals for between-group differences in proportions were calculated using the Newcombe method. All p-values were two-sided, and a threshold of p < 0.05 was considered statistically significant.

## Results

### Demographic and clinical characteristics

A total of 449 patients with solitary ACR TI-RADS 5 thyroid nodules were included, comprising 416 in the non-isthmus group and 33 in the isthmus group. Cytological results were available for 144 of 449 nodules (32.1%), including 134 of 416 nodules in the non-isthmus group and 10 of 33 nodules in the isthmus group. No significant differences between groups in terms of sex distribution (female: 79.80% vs. 75.76%, p = .58), mean age (43 ± 11.57 vs. 43 ± 11.16 years, p = 1.00), or the prevalence of Hashimoto’s thyroiditis (21.63% vs. 18.18%, p = .64). The mean duration of AS was comparable between groups (47 ± 23.00 vs.48± 24.50 months, p = .82). In terms of clinical outcomes, patients in the isthmus group were more likely to remain under ongoing surveillance (90.91% vs. 76.20%), whereas the non-isthmus group showed higher rates of loss to follow-up (20.91% vs. 9.09%). These differences did not reach statistical significance (p = .08) (**Table 1**).

**Table 1:** Demographic and clinical profiles of the non-isthmic and isthmic groups.

Characteristic	Category	Non-Isthmus group (n=416)	Isthmus group (n=33)	*p*-value
Sex	Female	332 (79.80%)	25 (75.76%)	0.58
	Male	84 (20.19%)	8 (24.24%)	
Age (year)		43 ± 11.57	43 ± 11.16	1.00
HT	Yes	90 (21.63%)	6 (18.18%)	0.64
	No	326 (78.37%)	27 (81.82%)	
Mean AS Time (months)		47 ± 23.00	48 ± 24.50	0.82
Outcomes after AS	Ongoing AS	317 (76.20%)	30 (90.91%)	0.08
	Lost to follow-up	87 (20.91%)	3 (9.09%)	
	Ablation	5 (1.20%)	0 (0%)	
	Surgery	7 (1.68%)	0 (0%)	

MD, maximum diameter; HT, Hashimoto’s thyroiditis; AS, Active surveillance.

### Baseline nodule characteristics

While the initial mean maximum diameter did not differ significantly between groups (5.55 ± 2.00 mm vs. 4.83 ± 2.69 mm, p = .06), the final maximum diameters remained comparable (p = .16). However, the non-isthmus group had greater baseline volume (79.69 ± 370.43 mm³ vs. 65.28 ± 60.01 mm³, p = .02) and larger final volume (107.08 ± 644.37 mm³ vs. 70.10 ± 78.12 mm³, p = .01). Bethesda cytology classifications showed a significantly higher rate of Bethesda category VI (malignant) nodules in the isthmus group (80.00% vs. 46.27%, p <.001). Although the isthmus group had a higher *BRAF* mutation rate (85.71% vs. 69.70%), this was not statistically significant (p = .67), and no *TERT* promoter mutations were detected in either group (**Table 2**).

**Table 2:** Nodule characteristics in the non-isthmic and isthmic groups.

Features	Category	Non-Isthmus group (n=416)	Isthmus group (n=33)	*p*-value
Mean MD (mm)	Initial Follow-up	4.83 ± 2.69	5.55 ± 2.00	0.06
	Last follow-up	5.01 ± 3.06	5.55 ± 2.01	0.16
Mean volume (mm³)	Initial follow-up	79.69 ± 370.43	65.28 ± 60.01	0.02^*^
	last Follow-up	107.08 ± 644.37	70.10 ± 78.12	0.01^*^
Bethesda category	I	5/134 (3.73%)	0/10 (0%)	0.37
	II	10/134 (7.46%)	0/10 (0%)	
	III	26/134 (19.40%)	0/10 (0%)	
	IV	2/134 (1.49%)	0/10 (0%)	
	V	29/134 (21.64%)	2/10 (20.00%)	
	VI	62/134 (46.27%)	8/10 (80.00%)	
*BRAF* mutation status	Positive	69/99 (69.70%)	6/7 (85.71%)	0.67
	Negative	30/99 (30.30%)	1/7 (14.29%)	
*TERT* mutation status	Positive	0	0	1.00
	Negative	68 (100%)	6 (100%)	

MD, maximum diameter; ACR TI-RADS, The American College of Radiology Thyroid Imaging Reporting and Data System. * indicates a statistically significant difference between the groups (P < 0.05).

### Disease progression during active surveillance

**Figure 6:**
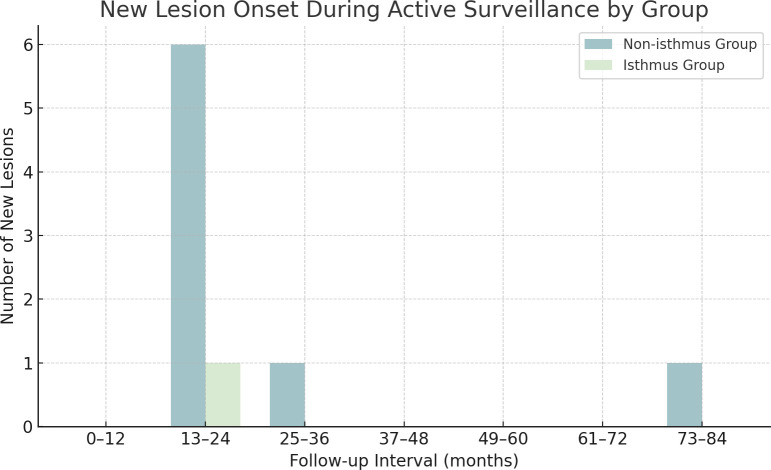
Time distribution of newly developed suspicious thyroid nodules during active surveillance in isthmic and non-isthmic groups. Bar graph illustrates the onset timing of new suspicious nodules among patients undergoing active surveillance, stratified by anatomical location. In the non-isthmus group, 75% (6/8) of new lesions emerged between 13 and 24 months, with additional cases at 34 and 77 months. In the isthmus group, a single new lesion occurred at 19 months.

Multiple progression indicators were evaluated separately and are reported descriptively in **Table 3**. A maximum diameter increase of ≥3 mm occurred in 3.03% of patients in the isthmus group (1/33; 95% CI 0.08%-15.76%) and in 2.64% of patients in the non-isthmus group (11/416; 95% CI 1.33%-4.68%), with an absolute risk difference of 0.39 percentage points (95% CI -2.83 to 12.73; p = .60). A ≥50% increase in volume occurred in 30.30% of patients in the isthmus group (10/33; 95% CI 15.59%-48.71%) and in 24.52% of patients in the non-isthmus group (102/416; 95% CI 20.46%-28.95%), with an absolute risk difference of 5.78 percentage points (95% CI -7.86 to 23.26; p = .53). Directional growth indicators, defined by changes of ≥3 mm in one, two, or all three orthogonal diameters, also showed no significant between-group differences. Newly developed lesions occurred in 3.03% of patients in the isthmus group (1/33; 95% CI 0.08%-15.76%) and in 1.92% of patients in the non-isthmus group (8/416; 95% CI 0.83%-3.75%), with an absolute risk difference of 1.11 percentage points (95% CI -1.98 to 13.43; p = .50). Among the eight newly developed lesions in the non-isthmus group, six occurred between 13 and 24 months, and the remaining two occurred at 34 and 77 months; the single newly developed lesion in the isthmus group occurred at 19 months (**Figure 6**). Lymph node metastasis occurred in none of the patients in the isthmus group (0/33; 95% CI 0.00%-10.58%) and in 2.16% of patients in the non-isthmus group (9/416; 95% CI 0.99%-4.07%), with an absolute risk difference of -2.16 percentage points (95% CI -4.06 to 8.31). A composite endpoint occurred in 1 of 33 patients in the isthmus group (3.03%, 95% CI 0.08%-15.76%) and in 17 of 416 patients in the non-isthmus group (4.09%, 95% CI 2.40%-6.46%), with an absolute risk difference of -1.06 percentage points (95% CI -4.49 to 11.33; p = 1.00).

**Table 3:** Comparison of progression indicators and subgroup analysis between the non-isthmic and isthmic groups.

Outcome	Isthmus group, n/N (%) [95% CI]	Non-isthmus group, n/N (%) [95% CI]	Absolute risk difference, percentage points [95% CI]	P value
MD Increase ≥3 mm	1/33 (3.03) [0.08-15.76]	11/416 (2.64) [1.33-4.68]	0.39 [-2.83 to 12.73]	.60
Volume Increase ≥50%	10/33 (30.30) [15.59-48.71]	102/416 (24.52) [20.46-28.95]	5.78 [-7.86 to 23.26]	.53
Longitudinal diameter Increase ≥3 mm	0/33 (0.00) [0.00-10.58]	10/416 (2.40) [1.16-4.38]	-2.40 [-4.37 to 8.08]	1.00
Anteroposterior diameter Increase ≥3 mm	0/33 (0.00) [0.00-10.58]	6/416 (1.44) [0.53-3.11]	-1.44 [-3.11 to 9.01]	1.00
Transverse diameter Increase ≥3 mm	1/33 (3.03) [0.08-15.76]	4/416 (0.96) [0.26-2.44]	2.07 [-0.83 to 14.37]	.32
Any two diameters Increase ≥3 mm	0/33 (0.00) [0.00-10.58]	4/416 (0.96) [0.26-2.44]	-0.96 [-2.45 to 9.48]	1.00
All three diameters Increase ≥3 mm	0/33 (0.00) [0.00-10.58]	2/416 (0.48) [0.06-1.73]	-0.48 [-1.74 to 9.95]	1.00
New Lesion	1/33 (3.03) [0.08-15.76]	8/416 (1.92) [0.83-3.75]	1.11 [-1.98 to 13.43]	.50
LNM	0/33 (0.00) [0.00-10.58]	9/416 (2.16) [0.99-4.07]	-2.16 [-4.06 to 8.31]	1.00
Distant Metastasis	0/33 (0.00) [0.00-10.58]	0/416 (0.00) [0.00-0.88]	0.00 [-0.91 to 10.43]	1.00
Composite endpoint	1/33 (3.03) [0.08-15.76]	17/416 (4.09) [2.40-6.46]	-1.06 [-4.49 to 11.33]	1.00

MD, maximum diameter; LNM, lymph node metastasis. Composite endpoint included newly developed lesions, LNM, or distant metastasis. Absolute risk difference was calculated as the proportion in the isthmus group minus the proportion in the non-isthmus group. Group-specific 95% confidence intervals were calculated using the Clopper-Pearson exact method, and 95% confidence intervals for between-group differences were calculated using the Newcombe method. P values for individual progression indicators should be interpreted as exploratory because multiple individual outcomes were assessed in addition to the composite endpoint.

## Discussion

This multicenter retrospective cohort study is, to our knowledge, the first to compare progression outcomes during active surveillance between isthmic and non-isthmic highly suspicious thyroid nodules. Observed differences in progression indicators were not statistically significant, but the results should be interpreted cautiously due to sparse events and multiplicity. Within these limitations, active surveillance may be feasible for selected isthmic nodules under careful monitoring.

AS has been widely validated in low-risk PTC, particularly for nodules ≤10 mm in maximum diameter. Over time, its application has expanded to include selected nodules measuring 10–20 mm without high-risk features (28, 30–33). A ≥3 mm increase in maximum diameter remains the most widely adopted threshold for defining disease progression in large-scale studies, particularly in East Asia. Although this definition may be considered conservative, its consistency across studies enhances comparability and supports its continued use in observational cohorts like ours (30, 33–35).

Isthmic nodules have often been regarded as less suitable for active surveillance because of their proximity to the anterior capsule and trachea, which may raise concern for earlier capsular involvement or extrathyroidal extension (15). Consequently, some surveillance protocols have excluded isthmic nodules or favored earlier intervention. In the present study, however, isthmic location was not associated with a higher rate of structural progression, which is in line with emerging evidence from surgical and ablation cohorts suggesting that isthmic location alone may not indicate more aggressive behavior (16, 18, 36–39). Although maximum diameter was comparable between the two groups at baseline and at the end of follow-up, non-isthmic nodules showed larger tumor volumes. This discrepancy highlights an important limitation of using maximum diameter alone to assess interval growth during surveillance. Changes in a single axis may underestimate multidirectional or asymmetric enlargement, whereas volume incorporates all three orthogonal dimensions and may better reflect overall tumor expansion. At the same time, volume estimation based on the ellipsoid formula remains sensitive to measurement variability and may be less reliable for irregularly shaped nodules. For this reason, we additionally evaluated directional growth indicators based on changes in one, two, or three orthogonal diameters as descriptive measures of structural change during surveillance. Although these indicators did not differ significantly between groups, they should be interpreted as exploratory descriptors of growth pattern rather than as confirmatory endpoints.

The incidence of newly developed suspicious lesions was low in both groups (3.03% in the isthmus group vs. 1.92% in the non-isthmus group; p = .50). In the non-isthmus group, 6 of 8 new lesions (75.0%) developed between 13 and 24 months, and the remaining 2 appeared at 34 and 77 months. The single case in the isthmus group occurred at 19 months. These findings suggest that new lesions were mostly clustered within the first two years of follow-up, underscoring the importance of intensified surveillance during the early and intermediate phases of AS. It is also important to emphasize that the emergence of a new suspicious lesion does not necessarily indicate progression of the original index nodule, but may reflect the multifocal nature of papillary thyroid carcinoma. Therefore, interpretation of this indicator should be approached with caution in the context of AS.

Although the isthmus group had higher proportions of ACR TI-RADS 5 and Bethesda category VI nodules, disease progression rates were not significantly different. This suggests that sonographic and cytologic features alone may not fully predict structural progression during AS, particularly when patient preferences and physician discretion influence management decisions. In our institution, isthmic location is often perceived as a potential high-risk feature. Nevertheless, many patients opted for continued AS even when nodules were anatomically adjacent to critical structures. This variability underscores the complex interplay between biological, clinical, and behavioral factors in real-world AS implementation.

In this study, cytological confirmation was available only in a minority of cases. Accordingly, this cohort should be interpreted primarily as a real-world active surveillance cohort of highly suspicious ACR TI-RADS 5 nodules, rather than a conventional biopsy-confirmed papillary thyroid microcarcinoma cohort. Although this differs from most published active surveillance series, it reflects a pragmatic clinical scenario in which some patients with highly suspicious ultrasound findings choose surveillance without prior FNA.

Additionally, although cytological (Bethesda category) and molecular (*BRAF* mutation) data were available for a subset of patients, the sample size was insufficient to support robust subgroup analyses. For instance, it remains unclear whether nodules with Bethesda category VI or *BRAF*-positive status are more likely to exhibit disease progression during AS. Future studies with larger cohorts and more complete molecular profiling are warranted to explore whether these factors independently influence the behavior of isthmic or non-isthmic nodules.

The ACR TI-RADS is one of the most widely adopted ultrasound-based risk stratification systems globally. Its diagnostic criteria—solid composition, marked hypoechogenicity, microcalcifications, irregular margins, and taller-than-wide shape—are consistent with those used in other major systems, including EU-TIRADS, K-TIRADS, and C-TIRADS (**Table 4**). Therefore, our findings may serve as a useful reference for clinicians across diverse diagnostic frameworks, particularly when evaluating nodules in anatomically sensitive locations such as the thyroid isthmus.

**Table 4:** Comparative overview of major thyroid nodule ultrasound risk stratification systems (C-TIRADS, ACR TI-RADS, EU-TIRADS, and K-TIRADS).

Dimension	ACR TI-RADS	EU-TIRADS	K-TIRADS	C-TIRADS
Developer	ACR†	ETA‡	KSThR§	CMA*
Risk Stratification Categories	TR1-TR5	EU-TIRADS 1-5	K-TIRADS 1-5	C-TIRADS1, 2, 3, 4A, 4B, 4C, 5, 6
Classification Method	Scoring method (points assigned according to ultrasound features)	Pattern-based approach (classification based on ultrasound feature patterns)	Pattern-based approach (classification based on ultrasound feature patterns)	Counting method (based on number of suspicious ultrasound features)
Typical Suspicious Ultrasound Features	Solid composition, markedly hypoechoic, microcalcifications, irregular margins, vertical orientation	Markedly hypoechoic, irregular margins, microcalcifications, vertical orientation, irregular shape	Solid composition, markedly hypoechoic, microcalcifications, irregular margins, vertical orientation	Solid composition, markedly hypoechoic, microcalcifications, irregular margins, vertical orientation, extrathyroidal extension
FNA Recommendation Threshold	≥10 mm (TR5); ≥15 mm (TR4); ≥25 mm (TR3, selective)	≥10 mm (EU-TIRADS 5); ≥15 mm (EU-TIRADS 4); ≥20 mm (EU-TIRADS 3, not routinely recommended)	≥10 mm (K-TIRADS 5); ≥15 mm (K-TIRADS 4)	≥10 mm (4B and above); ≥15 mm (4A)
ACR TI-RADS 5 Equivalent in Other Systems	——	EU-TIRADS 6	K-TIRADS 5	4B

CMA, Chinese Medical Association; ^†^ACR, American College of Radiology; ^‡^ETA, European Thyroid Association; ^§^KSThR, Korean Society of Thyroid Radiology.

*CMA, Chinese Medical Association.

This study has several limitations. First, the retrospective design may have introduced selection bias and unmeasured confounding. Second, although the study was conducted across multiple centers, the isthmus subgroup remained relatively small and progression events were infrequent, resulting in wide confidence intervals and limited power to detect small-to-moderate between-group differences. In addition, because the boundary of the thyroid isthmus is not sharply demarcated on all transverse ultrasound images, nodules located near the predefined border of the isthmic zone may have been susceptible to classification uncertainty, which could have attenuated true location-related differences in progression risk and contributed to the observed similarity in outcomes. Third, despite a median follow-up of 47 months, longer observation is still needed to capture late progression events.

In this multicenter retrospective cohort, highly suspicious thyroid nodules (ACR TI-RADS 5) located in the isthmus were not associated with higher progression rates than non-isthmic nodules during a median follow-up of 47 months. These findings support the potential feasibility of active surveillance for selected isthmic nodules under careful monitoring. However, given the retrospective design, the relatively small size of the isthmus subgroup, the low number of progression events, and the resulting limited statistical power, definitive conclusions regarding equivalence or formal comparability with non-isthmic nodules cannot be drawn. Larger prospective studies are needed to further clarify the role of active surveillance in this setting.

## Data Availability

The raw data supporting the conclusions of this article will be made available by the authors, without undue reservation.
